# Developing the American College of Surgeons Quality Improvement Framework to Evaluate Local Surgical Improvement Efforts

**DOI:** 10.1001/jamasurg.2022.1826

**Published:** 2022-06-15

**Authors:** Clifford Y. Ko, Tejen Shah, Heidi Nelson, Avery B. Nathens

**Affiliations:** 1 Division of Research and Optimal Patient Care, American College of Surgeons, Chicago, Illinois; 2 Department of Surgery, University of California, Los Angeles, Los Angeles; 3 Department of Surgery, The Ohio State University, Columbus; 4 Department of Surgery, University of Toronto, Toronto, Ontario, Canada

## Abstract

This quality improvement study establishes an 8-component framework to assess surgical improvement efforts that are reported to the American College of Surgeons.

The American College of Surgeons (ACS) Quality Programs collect more than 3500 improvement efforts annually. These efforts are usually local (ie, occurring within a hospital) or small scale (ie, low resourced, low funded, or unfunded)^1^ and routinely conducted by frontline clinicians and clinical teams. We tried to identify an appropriate and adequate framework to evaluate these improvement efforts; however, no single framework for small-scale, clinician-led improvement efforts exists. Current available frameworks focus on investigative or research-based efforts, efforts led by improvement specialists, or large-scale (ie, resourced, funded) improvements. Herein we describe the development of the ACS Quality Improvement Framework, whose function is to evaluate small-scale surgical improvement efforts.

## Methods

In this quality improvement study, framework development was conducted by the 12-member ACS Quality Programs Advisory Improvement Committee, which consists of surgeon or ACS staff representatives from 7 ACS Quality Accreditation Programs (focusing on trauma, cancer, breast disease, rectal cancer, children’s surgery, bariatric surgery, and geriatric surgery). Each committee member has extensive experience in leading and supporting surgical improvement efforts. A review of published frameworks in 3 areas (improvement science, program evaluation, and implementation science^2,3,4,5^) was initially conducted to identify framework components and associated criteria thought to be applicable to small-scale surgical improvement efforts. With identified framework components, a nominal group technique was conducted^6^ with 3 rounds of iterative prototype development and pilot testing on a sample of improvement efforts. Each round prioritized development on 3 constructs: content validity, face validity, and feasibility. Prototype frameworks for each round were evaluated for feasibility using a split sample of ACS Quality Program improvement efforts. The final framework also underwent pilot testing for reliability by individual committee member evaluation.

## Results

The literature review identified 88 published frameworks and 51 components. The number of components was reduced to 25 based on content and face validity for small-scale settings. The first 2 rounds of nominal group evaluations identified 12 and 10 components, respectively, and were each informed by pilot testing of 15 different randomly selected improvement projects. Pilot tests assessed validity and feasibility for component (and criteria) selection; the final framework consisted of 8 components with 39 criteria in total (Table, Box). The final framework test for reliability resulted in 80% agreement between raters (Cohen κ, 0.60), signifying moderate agreement.

**Table.** Components for Prototype Frameworks 1 and 2 and Final Framework

Component	Prototype 1	Prototype 2	Final framework
Problem detailing	X	X	X
Goal specification	X	X	X
Strategic planning	X	X	X
Evaluation			
Process	X	X	X
Outcome	X	X	X
Cost	X	X	X
Knowledge acquisition	X	X	X
End-of-project decision-making	X	X	X
Data factors	X	X	
Stakeholder involvement	X	X	
Improvement team factors	X		
Contextual factors	X		

X indicates the components included in each prototype.

Box. **Final Framework Components and Criteria With Descriptions**

**Problem Detailing**

- Problem statement: defined problem statement presents a clinical reason to pursue the project
- Local issue: problem is known to be a local issue, not just a broader problem that may or may not be a local issue
- Local data: problem is defined with data describing the local problem (eg, local data)
- Significance of problem: significance of the problem is explained (eg, a systematic issue; statistical outlier; lifesaving, costly, unsafe practice; nonstandardized or variability in practice with substantial untoward effects)
- Patient facing: project involved patient input regarding problem delineation or detailing to be patient centered
- Improvement team input: project improvement team involved with defining and identifying problem
- Internal stakeholders: internal stakeholders involved in defining and identifying problem; can include individuals such as doctors and nurses who are not part of the project improvement team
- External stakeholders: stakeholders external to project team and internal stakeholders involved in defining and understanding problem (eg, payer, hospital leadership, consultants)

**Goal Specification**

- Specific: project goal is specific (ie, details specific outcomes, complications and part of care to be improved)
- Measurable: project goal is measurable (ie, lists metrics or data to be used to indicate if project will be successful in the outcome evaluation)
- Achievable: project goal is achievable (ie, the amount of improvement to be achieved is reasonable, being informed by project team and stakeholders)
- Relevance: project goal is relevant (ie, project is linked with applicability to patients and stakeholders)
- Timely: project goal is time driven (ie, the aim states the project will be completed within a specific time frame, such as within a year)

**Strategic Planning**

- Improvement planning: planning of the project that describes the strategy being undertaken (eg, use of checklist, protocol, educational program, or a combination)
- Strategic rationale: included in the project planning is the rationale for the chosen strategy (eg, why this strategy was chosen and why the chosen strategy will work for the identified problem in this setting; can include citing of evidence for the chosen strategy)
- Stakeholder involvement: stakeholder involvement and input into planning of the strategy (eg, chief executive officer support, patient involvement, surgeons, anesthesia, and nursing)
- Resources: information needed to operationalize the project (eg, who is undertaking each part of the effort, time required, and equipment needed)
- Data: use of data planned before start of project (eg, specifies the data to be used and how it will be used; may include metrics, collection method, analytics, and other data aspects)
- Probable limitations: probable or possible limitations, challenges, or hurdles identified a priori (during planning stages and before project begins)
- Contextual issues: identified to successfully fulfill the strategy (eg, addressing culture, engagement, training and education of patients, staff, or improvement team)

**Process Evaluation**

- Description of project execution: methods described, specifically of implementation strategy (ie, describes what was planned to do, what was executed)
- Evaluation during implementation: the implementation of the improvement effort is evaluated (eg, describes an assessment [formal or informal] during the improvement project focusing on how well the implementation was being undertaken from launch through completion)
- Problems encountered: the barriers and problems are identified throughout the project from launch to completion
- Changes undertaken: the changes undertaken in the method, implementation, strategy (other) in response to barriers and problems are identified and captured
- Data: used to evaluate processes (eg, protocol compliance and inadequate resources)
- Stakeholder involvement: achieved to inform evaluation of project processes, protocols, and strategies

**Outcome Evaluation**

- Data and analytics: used to measure effectiveness of the effort and achievement of project goals
- Outcome quantification: project outcome is quantified
- Goal achievement: improvement team determines whether the specified project goals were achieved fully or partially or were not achieved
- Biggest limitations: limitations in determining the outcome are identified in review of the entire effort after completion
- Unintended consequences: identified and captured
- Stakeholders: made aware of outcomes

**Cost Evaluation**

- Cost of project: costs are recorded (eg, any monetary or budget allotment and FTE, so it is known how much it might cost to replicate the project)
- Value: value of doing the project is known (eg, whether the outcome was worth the effort, either formal or informal)
- Stakeholders’ perspective: stakeholders assess value of the project, taking into account resources, processes, and outcomes

**Knowledge Acquisition**

- Lessons: lessons captured
- Current and future action: any actions mentioned regarding use (eg, sharing and publishing) knowledge acquired and lessons learned

**End-of-Project Decision-Making**

- Future actions: decision on whether the effort will continue as is, be expanded to other areas, continue only with revision, be stopped, or other
- Surveillance plans: plans for surveillance for recurrence of the problem are stated

FTE indicates full-time equivalent (salary).

## Discussion

We developed a quality improvement framework to evaluate surgical improvement efforts reported to the ACS. It was specifically developed for and tested on small-scale surgical improvement efforts collected by sites participating in ACS Quality Programs. The planned function for the framework is to evaluate improvement efforts submitted to the ACS—with 2 goals: (1) ascertain how well the efforts were conducted; and (2) identify gaps in execution, aiming to advance improvement efforts.

The framework is unique for 2 reasons. First, to our knowledge, it is the first that specifically focuses on small-scale efforts—meaning it purposely addresses the type of improvement efforts largely seen in surgery in which local efforts that are clinically important are also often low resourced, unfunded, occurring in single settings like a ward or unit, and conducted by busy frontline surgeons. Second, this framework eliminates issues more relevant to large-scale or investigative research efforts yet maintains core items, such as problem identification, identifying project aims, and use of data—all important to frontline efforts.

The framework also can be used to pragmatically guide conduct of a small-scale improvement effort—the planning, conducting, middle- and end-of-project evaluation, and reporting. Efforts are in progress to incorporate this framework into ACS Quality Programs to help frontline clinicians better execute improvement efforts, and we believe this framework will support these efforts.
